# A Tooth Growing in the Nose

**Published:** 1886-10

**Authors:** 


### A Tooth Growing in the Nose.

In the *Medical Record*, Dr. E. Hanson Griffin, of New York City, reports the following case:

"Patient, G. I——, aged thirty-two, applied to the Bellevue Throat Clinic, complaining of a sore throat and a pain in the back of the head of two days' duration.

"An examination of his throat showed that there was a cleft of both the soft and hard palate. In examining the upper gum I noticed the root of the second incisor tooth in the tissue, while an examination of the nose showed the tooth in a perfect state of development projecting from the floor of the nostril upward into the nasal cavity. He said the tooth had been there for years, and give him no trouble. He never remembered suffering any particular pain when it was growing, in fact did not know anything was the matter with his nose till one day while putting his finger into it he noticed something hard, and looking into a mirror found it to be a tooth. As it gave him no trouble, he did not apply to a physician. He does not think the tooth has grown any of late years. He was a twin, but his sister's throat was in perfect condition, and her nose free from any defect. The patient was opposed to any operation for the removal of the tooth, and as his sore throat was of a trivial character and not dependent upon this trouble, I did not urge a destruction of one of nature's whims."

